# Virtual pathway explorer (viPEr) and pathway enrichment analysis tool (PEANuT): creating and analyzing focus networks to identify cross-talk between molecules and pathways

**DOI:** 10.1186/s12864-015-2017-z

**Published:** 2015-10-14

**Authors:** Marius Garmhausen, Falko Hofmann, Viktor Senderov, Maria Thomas, Benjamin A. Kandel, Bianca Hermine Habermann

**Affiliations:** CECAD Research Center, Joseph-Stelzmann-Str. 26, 50931 Cologne, Germany; Gregor Mendel Institute of Molecular Plant Biology, Austrian Acacdemy of Sciences, Vienna Biocenter (VBC), Dr. Bohr-Gasse 3, 1030 Vienna, Austria; Dr. Margarete Fischer-Bosch Institute of Clinical Pharmacology and University of Tübingen, Auerbachstr. 112, 70376 Stuttgart, Germany; Research Group Computational Biology, Max Planck Institute of Biochemistry, Am Klopferspitz 18, 82152 Martinsried, Germany; Present address: Pensoft Publisher, 1700 Sofia, Bulgaria; Present address: Hain Lifescience GmbH, Hardwiesenstr. 1, 72147 Nehren, Germany

**Keywords:** Focus network, Disease state, Shortest path algorithm, Node neighborhood, Pathway enrichment

## Abstract

**Background:**

Interpreting large-scale studies from microarrays or next-generation sequencing for further experimental testing remains one of the major challenges in quantitative biology. Combining expression with physical or genetic interaction data has already been successfully applied to enhance knowledge from all types of high-throughput studies. Yet, toolboxes for navigating and understanding even small gene or protein networks are poorly developed.

**Results:**

We introduce two Cytoscape plug-ins, which support the generation and interpretation of experiment-based interaction networks. The virtual pathway explorer viPEr creates so-called focus networks by joining a list of experimentally determined genes with the interactome of a specific organism. viPEr calculates all paths between two or more user-selected nodes, or explores the neighborhood of a single selected node. Numerical values from expression studies assigned to the nodes serve to score identified paths. The pathway enrichment analysis tool PEANuT annotates networks with pathway information from various sources and calculates enriched pathways between a focus and a background network. Using time series expression data of atorvastatin treated primary hepatocytes from six patients, we demonstrate the handling and applicability of viPEr and PEANuT. Based on our investigations using viPEr and PEANuT, we suggest a role of the FoxA1/A2/A3 transcriptional network in the cellular response to atorvastatin treatment. Moreover, we find an enrichment of metabolic and cancer pathways in the Fox transcriptional network and demonstrate a patient-specific reaction to the drug.

**Conclusions:**

The Cytoscape plug-in viPEr integrates –omics data with interactome data. It supports the interpretation and navigation of large-scale datasets by creating focus networks, facilitating mechanistic predictions from –omics studies. PEANuT provides an up-front method to identify underlying biological principles by calculating enriched pathways in focus networks.

**Electronic supplementary material:**

The online version of this article (doi:10.1186/s12864-015-2017-z) contains supplementary material, which is available to authorized users.

## Background

The integration and biological interpretation of large-scale datasets is currently one of the main challenges in bioinformatics research. How can we extract meaningful information from a list of differentially regulated genes? One possibility to understand, how (co-)regulated genes relate to each other is to view them in the context of their physical, genetic or regulatory interactions: network-based analysis using data from protein-protein or regulatory interactions can open new perspectives for further experimental studies.

Quantitative values from a functional screen or a list of mutated genes identified in a cancer genomics study can be used to generate sub-networks from a large, biological interaction network. These sub- or focus networks can be termed ‘disease’ or ‘state’ networks, as they describe the modules in the cell or the organism, which are affected by a certain experimental condition or by a particular disease. This approach has for instance been employed by software like the database and web-tool String [1] or the command-line based tool Netbox [2].

Focus networks can also be created based on a specific biological question: how are two specific proteins - or two groups of proteins - connected with each other? This approach allows an even more biologically focused view on the changes in the cellular network under different conditions.

Focus networks allow us moreover to understand the cross-talk between two molecules or pathways, which in this context is defined by all paths between two proteins or two groups of proteins.

Typically, some form of shortest-path algorithm like Dijkstra’s algorithm [3] is used to create sub-networks between two or more nodes. The numeric values from functional genomics studies are used to score paths between two nodes. Methods like Pathfinder [4] or the Reactome browser [5] have implemented this functionality of connecting two molecules with each other within a biological network. Both tools use numeric values also to visualize regulatory changes that take place during state changes of the cell/organism under study.

Focus networks can be further enriched using Gene Ontology (GO) terms [6] or pathways from different sources to provide additional functional information for data interpretation. GO biological processes can also be used to explore cross-connections between two or more pathways and find missing pathway components. This provides a more integrative view of a biological network.

The drug family of the statins is currently widely used to lower cholesterol levels in the treatment of hypercholesterolemia. Statins, which act as HMG-CoA (3-hydroxy-3-methylglutaryl–coenzyme A) reductase (HMGCR) inhibitors, prevent the production of cholesterol by inhibiting the biosynthesis of isoprenoids and sterols in the mevalonate pathway [7]. However, statins are known to have a variety of side effects, including muscle adverse effects, liver damage, cognitive impairment, cancer progression or diabetes mellitus [8–11]. Functional genomics studies of statin-treated cell systems indicate extensive changes of expression levels upon drug treatment (see for instance [12–20]). The detailed analysis of these transcriptional changes should therefore lead to a better understanding of the functions and pleiotropic effects of statins.

In this study, we re-analyzed the time-course expression data from atorvastatin-treated, primary human hepatocytes from six different patients published in a previous study [20]. We focused our analysis on determining the regulation of downstream genes from statin drug targets as defined in STITCH [21]. We were especially interested in addressing the following issues: 1) How do statin targets and differentially regulated genes relate to each other? 2) Which pathways are affected upon statin treatment? 3) How does the dynamics of the neighborhood of specific proteins change after statin treatment?

In order to answer those questions, we have developed two Cytoscape plug-ins that work together: viPEr, the virtual Pathway Explorer, creates focus interaction networks by connecting two or more nodes with each other. It applies user-provided expression data to score paths between two nodes and thus limits the network to functionally relevant paths. The Cytoscape plug-in PEANuT (Pathway Enrichment ANalysis Tool) upgrades interaction networks with pathway information and identifies enriched pathways in focus networks.

We have applied our toolbox to re-analyze the expression data from atorvastatin-treated, primary human hepatocytes and found that the transcription factors FOXA1, 2 and 3 are important regulatory players in atorvastatin response.

## Implementation

### viPEr

viPEr was written in Java as a Cytoscape plug-in. The basis of all functions is a recursive method, which iterates through the members (nodes) of all paths emanating from a selected node. The step depth is influenced by two parameters: 1) the maximum number of steps allowed (set by the user). 2) the numerical values of the nodes. We used the log2fold expression changes of atorvastatin treated primary hepatocytes described in [20] as numerical values.

viPEr can be accessed under: http://sourceforge.net/projects/viperplugin/

viPEr has three main search options:

1. ‘**A to B**’: ‘**A to B**’ connects two selected nodes with each other. We refer to the paths between nodes A and B as cross-talk. Mathematically, we define cross-talk as all paths between two nodes (x1, *x*2), where a single node in a path can only be passed once. The result is a focus network, which is determined by the maximum number of steps allowed between the start and the target node. The search is stopped when the target node is reached or the maximum number of steps is exceeded. Only if the target has been found, a path is stored, scored and displayed in the results tab. The focus network is created based on all nodes that are present in all stored paths. The connecting edges are taken from the original network. Therefore, all known interactions between the subset of nodes are included in the newly created focus network.
  Scoring of paths is done using the following equation:
  $$ Score=\frac{\#\kern0.5em  of\kern0.5em  differently\kern0.5em  regulated\kern0.5em  nodes\kern0.5em \in \kern0.5em  path}{(pathlength)^2} $$
  The p-values for discovered paths in focus networks are calculated based on the cumulative probability of the hypergeometric distribution to find k or more differentially expressed genes in a path of length n.
2. **‘connecting in batch’**: similarly to the ‘**A to B**’ search, two groups of nodes can be connected using the ‘**connection in batch**’ function. For every node in the start list **A**, the recursive search is computed towards every node in the target list **B**. A results tab with scored paths is not created in this case.
3. **‘environment search’**: The third option is to explore the regulated proximity of a single node using the ‘**environment search**’. Just one starting node is selected in this case. Mathematically, we define the environment search as follows: a network is calculated from all outgoing paths of length *l* from x1, where every node is allowed to be passed only once per path and all paths with at least two consecutive node scores below threshold *t* have been removed. The iteration through emanating paths is carried out until the allowed maximum search depth is reached. When exploring the neighborhood of a single node, the numerical data are used to select paths radiating from the selected node. Paths, in which at least two consecutive nodes are not differentially expressed, are removed from the resulting neighbor focus network. Thus, only paths that contain differentially regulated nodes are considered for the environment search, though single unregulated linker nodes are allowed. The resulting network is referred to as a neighbor focus network.

#### Using viPEr

**Fig. 1:**
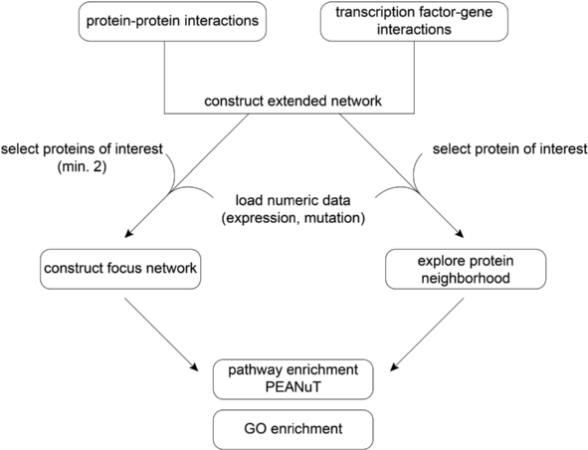
Workflow for creating focus networks. Workflow of viPEr in creating focus networks between two nodes/two groups of nodes, or in exploring the neighborhood of a single node of interest. The user must select two nodes or group of nodes for creating a focus network. A single node is selected when exploring the neighborhood. Numerical data (for instance from an expression screen) must be added to the network for scoring paths of a focus network and for creating a neighbor focus network from a single node. In both cases, the user selects the search depth. After creating the focus network, the network can for instance be explored by using and visualizing GO-terms. PEANuT is used to find and visualize enriched pathways

Starting from any existing network supplemented with expression data, the user has to select the attribute field containing the expression information. A slider is automatically set to the respective range of expression values. After adjusting the slider to the desired expression range, different options are available in the workflow (see Fig. 1).

1. **‘A to B’**
  This function executes the path search algorithm between two selected nodes. The result is a focus network of all identified paths of a certain length between two nodes. The user selects the length (step-size) of the calculated paths. All interconnecting edges are added to the focus network. A result list, which includes every discovered path between the nodes, is located on the right side of the screen. This list shows all paths, their respective members and the assigned score as described above. The score can be used to further reduce the focus network or simply to visualize specific paths.
2. **‘connecting in batch’**
  Two groups of nodes can be connected in the **‘connecting in batch’** function of viPEr. The same algorithm is used as in the **‘A to B’** search, except that all paths between all members of a start list and a target list are computed. This algorithm can be applied to detect cross talk between two pathways, two protein complexes or two hit lists from different experiments. Three buttons have to be used for the **‘connecting in batch’** search: 1) a start protein list has to be defined by selecting all starting nodes and pressing the **‘select start protein list’**; 2) the target protein list has to be selected accordingly and confirmed by pressing the **‘select target protein list’** button; 3) the button **‘start connection in batch’** executes the search.
3. **‘environment search’**
  In case only a single protein of interest exists, the algorithm can be used to observe the dynamics of expression in the environment of this protein using the **‘environment search’**. A single node is selected and the search is executed with the button **‘environment search’**. All regulated nodes within a certain step size of the selected protein give rise to the neighbor focus network.

### PEANuT

PEANuT (Pathway Enrichment ANalysis Tool) is a Cytoscape plug-in designed to annotate protein interaction networks with biological pathway information and to identify enriched pathways in focus networks. The interactome of the organism denotes the background network. Next to visualizing enriched pathways in the focus networks, the results can be exported as a tab delimited file.

PEANuT can be accessed under: http://sourceforge.net/projects/peanut-cyto and was implemented in Java.

The user can choose between the three databases ConsensusPathDB (http://consensuspathdb.org/, [22]), Pathway Commons (http://www.pathwaycommons.org/, [23]) and Wikipathways (http://www.wikipathways.org/ [24]) to annotate the network. While ConsensusPathDB requires Entrez gene IDs as input, Pathway Commons and Wikipathways require UniProt accession numbers. Annotation of nodes with these IDs can be done within Cytoscape using for instance the plug-in CyThesaurus [25].

ConsensusPathDB and Pathway Commons contain pathway data collected from publicly available pathway databases (e.g., Reactome [26], KEGG [27]; see the respective homepages for more information). WikiPathways is a database based on the ‘wiki principle’ and provides an open platform dedicated to collaborative registering, reviewing and curation of biological pathways.

While Pathway Commons and WikiPathways work with a wide variety of organisms, ConsensusPathDB is specialized on human, mouse and yeast pathways. When the user chooses to annotate his network of interest with ConsensusPathDB data, he can additionally import directed interactions from KEGG to increase the amount of vertex degrees, enabling more complex path searches using viPEr.

Information from Pathway Commons is accessed over their web service. Flat files from the ConsensusPathDB and WikiPathways webpages are downloaded via the Apache Commons IO library (http://commons.apache.org/proper/commons-io/) and Cytoscape internal downloader classes.

Once downloaded, ConsensusPathDB and WikiPathways can be used offline, while Pathway Commons requires internet access. Network annotation with Pathway Commons is slower, as it depends on the load and availability of the host server, as well as internet connection speed.

The probability value for the pathway enrichment in the focus network is determined using the Apache Commons Math library (http://commons.apache.org/proper/commons-math/) to calculate the cumulative probability of a hypergeometric distribution. Multiple testing correction is achieved by applying either Bonferroni [28] or Benjamini-Hochberg [29] correction.

PEANuT has three sub-menus:

1. **‘find pathways’**: the find pathways sub-menu annotates the networks in Cytoscape with pathway data. Networks can be labeled using more than one pathway resource by re-using the sub-menu with different pathway selections.
2. **‘show pathway statistics’**: the **‘show pathway statistics’** sub-menu calculates enriched pathways in a selected focus network. The user has to select the focus network of interest, the background network and choose a p-value cut-off. Enriched pathways can be selected for visualization and downloaded as a tab-delimited file.
3. **‘download/update dependencies’**: this sub-menu is used to download pathway information for network annotation. It needs to be run before using PEANuT the first time and should be run regularly to update pathway information.

#### Using PEANuT

After installing PEANuT in Cytoscape by placing the plug-in in the Cytoscape plug-in folder, the tool can be accessed via the plug-in menu. The sub-menus are used as follows:

1. ‘**find pathways**’
  This sub-menu allows the user to start the software and annotate the network(s) of choice with pathway data. In a simple dialog the user can select between three different databases: ConsensusPathDB, Pathway Commons or WikiPathways. The user can select different options for each database depending on preferences (such as import of directed interactions from KEGG). Annotating the network with more than one database is possible by subsequently re-running this sub-menu with another selected database.
2. **‘show pathway statistics’**
  When the user has finished annotating the network, clicking on the ‘**show pathway statistics**’ sub-menu will invoke a table, two combo boxes, two text fields and several buttons. In one of the text fields the user can select the p-value cut-off for the enrichment calculations. The combo boxes permit the user to select the focus and background network. Once the results have been calculated the user can export the results as a tab delimited file or search the table for pathways via the text box. The user can select each pathway and highlight the members of the pathways in the focus network by clicking on **‘select’**.
3. **‘download/update dependencies’**
  In this sub-menu, dependencies can be used to download/update information from ConsensusPathDB and WikiPathways. **‘download/update dependencies’** needs to be executed when first using the plug-in. It is good practice to refresh the dependencies from time to time by clicking on the ‘**download all**’ button.
  For more information on how to use PEANuT, see our wiki http://sourceforge.net/p/peanut-cyto/wiki/Home/.

### Analysis of expression data

#### Cluster analysis

We used preprocessed data from Schröder et al. [20], which have already been normalized and filtered and repeated the clustering step described in the original publication. The patient data used here were part of a large-scale study originally published in [20] and deposited at the Gene Expression Ominbus [30] resource (Accession number GSE29868). Tissues and corresponding blood samples of the original study were taken with written informed consent from donors. The study was furthermore approved by the ethics committees of the medical faculties of the Charite, Humbold University and University of Tübingen. Before clustering, the data were log2 transformed and filtered for probes, which were at least 1.7 fold differentially expressed in at least 2 patients. With these 12,554 probes EDISA clustering was performed, using the same parameters as in Schröder et al. [20], with tG of 0.05 and tC of 0.25.

#### Creating the PP/TFG Interaction data

The basis for biologically meaningful network analysis is a reliable interaction network with high confidence. To accomplish this goal, we allowed only high confidence interaction data. No predicted interactions or interactions based on co-expression were included. As source for protein-protein interactions, we chose Pathway Commons [23] due to its large number of curated interactions and the simple format, which can be directly loaded in Cytoscape. Likewise, only experimentally verified transcription factor – gene interactions from TRANSFAC® were included in the network. Confidence scores from TRANSFAC® were used to identify reliable regulatory interactions.

#### GO and pathway enrichment of gene lists and networks

To analyze GO and pathway enrichment of focus networks, the official gene symbols (HGNC) were submitted to DAVID [31]. Additionally, the GO annotation for all nodes was added to the network via Cytoscapes’ built-in function.

#### Clustering of patient data using Cluster3

Cluster3 [32] was used to cluster patient data of the FoxA1 neighbor focus network, as well as the 24 h time point of the probe ID of all patients. Standard parameters and average linkage clustering were chosen. The FoxA1 network was analyzed for presence (1) or absence (0) of a node. In case of up-regulation, the value (2) was assigned, while down-regulated nodes were assigned the value (−2) (see Additional file 1: Table S12).

## Results and discussion

### Expression analysis of hepatocyte time-course experiment

In a first analysis, we found a total of 12.554 differentially expressed probes in at least two patients irrespective of time-points (Additional file 1: Table S1). We processed these genes with EDISA 3D clustering [33], which resulted in 902 differentially regulated genes (823 non-redundant genes) that were grouped into 24 clusters (Additional file 1: Tables S2 and S3). Further analysis was performed with the non-redundant version of this gene set.

### GO and pathway enrichment

We submitted the processed gene list to DAVID [31] for functional annotation and enrichment analysis (Additional file 1: Tables S4 and S5). DAVID was chosen for its extended functionality beyond enrichment of Gene Ontology terms. Primarily, basic cellular and metabolic processes were enriched, such as amino acid or nucleic acid metabolism. In addition, several pathways and processes related to cellular cholesterol and lipid -metabolism and -homeostasis were enriched. Based on these results we could however not make any conclusions on the regulatory or transcriptional network involved in atorvastatin response.

### Focus network analysis

Next, we used focus network analysis to extract more information from the data. We decided to use the interaction network provided by Pathway Commons [23] as the basic protein-protein interaction network, as it contains information from more than one primary source (including BioGRID, IntAct or Reactome). Given the type of data in protein interaction databases, we reasoned that we additionally needed information on gene regulatory relationships: which transcription factors are able to regulate which gene set? We considered this information on transcription factor - gene interactions (TFGI) as essential to identify the gene regulatory networks that control the cellular response to statins at a transcriptional level. We retrieved TFGIs from TRANSFAC® and combined them with the protein-protein interactions (PPIs) from Pathway Commons to a single interaction network, which was used for all further analysis steps (see Fig. 1).

Next, we extracted the top ten high confidence primary drug targets of atorvastatin from the STITCH database [21] (Additional file 1: Table S6), all of which were present in our interaction network. We used the viPEr **‘connecting in batch’** function to connect the ten atorvastatin targets with the 823 differentially regulated, non-redundant genes we have identified with EDISA 3D clustering, using a step-size of two. This functionality of viPEr creates focus networks by searching for all paths between two proteins or two groups of proteins, up to a user-defined path length. The nodes are supplemented with expression values. These weights on the nodes are used to score all paths in the resulting focus networks. The score of a path is dependent on its length and the number of differentially regulated nodes it contains.

The resulting atorvastatin focus network contained 1107 nodes and 22029 edges (Additional file 2: Figure S1). Of those, 21516 were PP interactions and 513 came from TFG interactions. We next performed GO enrichment of the atorvastatin focus network using DAVID (Additional file 1: Table S7). We observed that with the proteins from the focus network, we found more terms relating to regulatory functions, such as signaling or transcriptional control than with the 823 differentially expressed genes alone.

We searched for enriched pathways in the atorvastatin focus network. To this end, we employed our pathway enrichment tool PEANuT on the focus network, taking the entire network of PPIs and TFGIs as a background. Using ConsensusPathDB [22] as pathway resource, PEANuT identified a total of 926 pathways, many of which were redundant. Notably, again mainly signaling-, as well as transcriptional regulation pathways were identified (Additional file 1: Table S8). We compared results of PEANuT with pathway enrichment results from DAVID (Additional file 1: Table S9). Though different pathway resources are available in DAVID, a similar set of pathways was enriched. Those included growth receptor signaling pathways such as the EGF-receptor-, VEGF-, Insulin- and Ras-signaling pathways, Interleukin signaling pathways, as well as cancer pathways. Due to the availability of the pathway interaction database (PID, [34]) of the ConsensusPathDB resource, however, more transcription factor pathways were identified with PEANuT.

### Involvement of the FoxA transcription factors in atorvastatin response

**Fig. 2:**
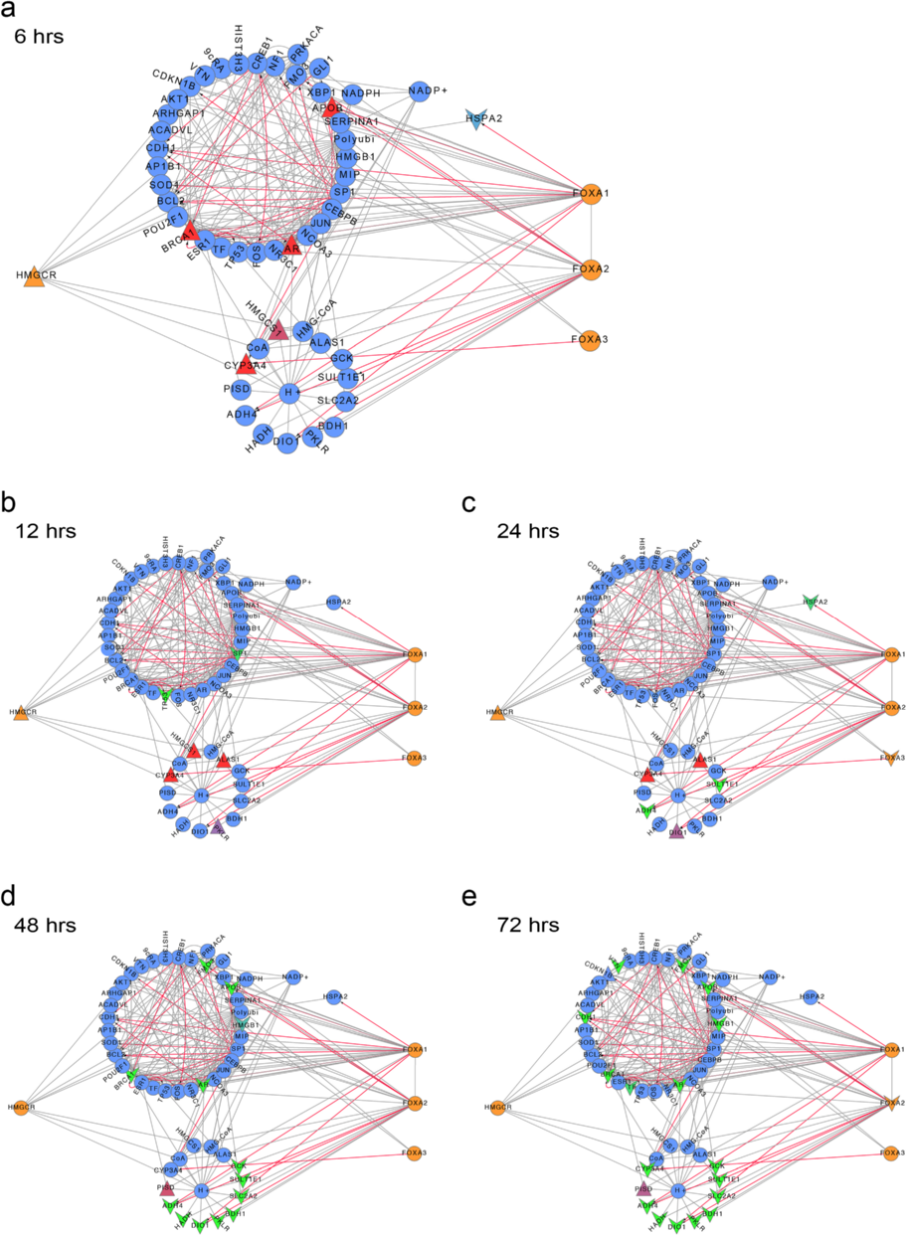
Focus networks between the primary atorvastatin target HMGCR and the transcription factors FoxA1, A2 and A3 of patient 65. Time course of patient 65 is shown at 6 h (**a**), 12 h (**b**), 24 h (**c**), 48 h (**d**) and 72 h (**e**). The focus networks were created using viPEr, with a maximal path length of 2 without considering the log2fold change: this allows direct comparison of time-course data. Up regulated nodes are shown as upward triangles, colored in red. Down regulated nodes are displayed as downward arrows, colored green. The Fox transcription factors are colored orange, as is the HMGCR. Protein-protein interactions have grey edges; edges of transcription factor gene interactions are colored red

In the list of enriched pathways, our attention was caught by the Forkhead box A transcription factor pathways. In adults, the Forkhead box A transcription factors FoxA1, FoxA2 and FoxA3 are expressed in liver, pancreas and adipose tissue, where they regulate gene expression of metabolic genes [35]. Two direct targets of atorvastatin are also targets of the FoxA transcription factors: FoxA1 and FoxA2 regulate ApoB [36–38], while Cyp3A4 is a target of FoxA3 [39]. Given our experimental set-up of atorvastatin-treatment of primary human hepatocytes and the fact that atorvastatin is primarily acting on cholesterol synthesis in the liver, we further focused our analysis on these two transcriptional pathways. We created a viPEr focus network between the primary atorvastatin target HMGCR and the three transcription factors FoxA1, A2 and A3 (Fig. 2) starting from the atorvastatin focus network with a maximal step size of two, without considering a fold-change.

We visualized expression changes over time and patients in the HMGCR-FoxA1/2/3 focus network. We first analyzed the expression changes over time from patient 65 in the focus network (Fig. 2). HMGCR itself is up-regulated at the first three time points, returning to normal expression values at the later two time points. Likewise, Cyp3A4 is up regulated at time points 6, 12 and 24, however becomes down regulated at 72 h.

Next, we were interested, whether the expression response to atorvastatin was similar in the six different patients and plotted HMGCR-FoxA1/2/3 focus networks for each patient at 12 h after treatment (Additional file 3: Figure S2). This representation of the data was especially informative as it illustrated the obvious differences in response to atorvastatin in the primary hepatocytes from the six patients. We observed that only donors 62, 65 and 79 showed somewhat overlapping regulatory responses at 12 h after treatment. The expression pattern of patient 67 was in many cases opposite to the first group (62, 65 and 79), while the hepatocytes from patients 80 and 81 showed milder responses to the drug.

### Interpreting the HMGCR-FoxA1/2/3 focus network

**Fig. 3:**
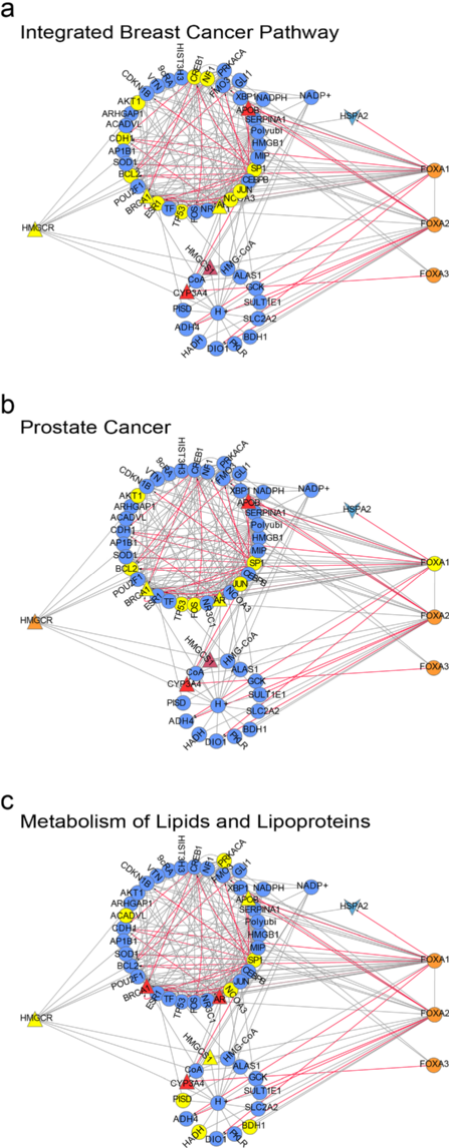
Enriched pathways identified by PEANuT on HMGCR/FoxA focus networks. Three pathways were chosen for display: the *Integrated Breast Cancer Pathway* (**a**), the *Prostate Cancer Pathway* (**b**) and the *Metabolism of Lipids and Lipoproteins Pathway* (**c**). Members of the respective pathways are highlighted in yellow. Up regulated nodes are shown as upward triangles, colored in red. Down regulated nodes are displayed as downward arrows, colored green. The Fox transcription factors are shown in orange, as is the HMGCR. Protein-protein interactions have grey edges; edges of transcription factor gene interactions are colored red

The focus network of HMGCR and the FoxA transcription factors can be divided in two parts (see Figs. 2 and 3). A small, tightly connected sub-cluster contains small molecules, as well as metabolic genes, while the larger sub-cluster contains signaling molecules, as well as transcription factors. HMGCR is directly connected to two proteins from this cluster: the cAMP dependent protein kinase alpha, PRKACA and Rho GTPase activating protein 1, ARHGAP1.

Especially FoxA1 is tightly integrated with other transcription factors from the large sub-cluster, including the transcription factors Sp1, Tp53, Fos, Jun or Brca1. It also binds several transcriptional co-activators.

Note that HMGCR expression is not directly regulated by any of the transcription factors in the network. The connections between the FoxA proteins and HMGCR are mostly due to transcriptional targets of the FoxA’s interacting with PRKACA and ARHGAP1.

Interestingly, statins have been implicated to have a preventive effect on different cancer types and to be beneficial for the treatment of several cancer types, among which are breast and prostate cancer [40–44].

Bcl2, a direct transcriptional target of FoxA1 [45], is also a direct interactor of the HMGCR interacting kinase PRKACA. Previous studies have shown that Bcl2 is down-regulated in response to statin-induced apoptosis ([46]; for a review on the involvement of Bcl2 in statin-response, see [47]); PRKACA has on the other hand been implicated in statin-resistance of tumors by phosphorylating the pro-apoptotic protein Bad (Bcl2 associated death receptor), thus allowing anti-apoptotic signaling [48].

In conclusion, cross-talk via PRKACA and ARHGAP1 is therefore one possible link between the mevalonate pathway and the FoxA transcriptional network, which could in part explain the effect of statins on apoptosis of cancer cells.

### Pathway enrichment analysis on focus networks using the Cytoscape plug-in PEANuT

We performed pathway enrichment analysis using PEANuT on the HMGCR-FoxA1/2/3 focus network (Additional file 1: Table S10). Again, many signaling, as well as metabolic pathways were enriched. In accordance with previous reports on statin-sensitivity of cancer cells [41, 42, 46, 49], several cancer pathways are enriched in our focus network. In Fig. 3, we illustrate the usefulness of PEANuT in visualizing components of enriched pathways in focus networks created with viPEr. We chose to highlight the two cancer pathways ‘***integrated breast cancer’*** (Wikipathways) and ‘***prostate cancer’*** (Wikipathways), as well as the pathway ***metabolism of lipids and lipoproteins*** (Reactome) in the HMGCR-FoxA1/2/3 focus network. Similar pathways were identified using DAVID (Additional file 1: Table S11).

### Exploring the molecular environment of a node using viPEr

In a next analysis step, we used viPEr’s functionality **‘environment search’** to explore the neighborhood of a single protein of interest. As a rule, only one linker node without differential expression is allowed in the **‘environment search’**. The user sets again the search depth by defining the step size. All paths that lack differentially expressed nodes are removed from the resulting neighbor focus network.

**Fig. 4:**
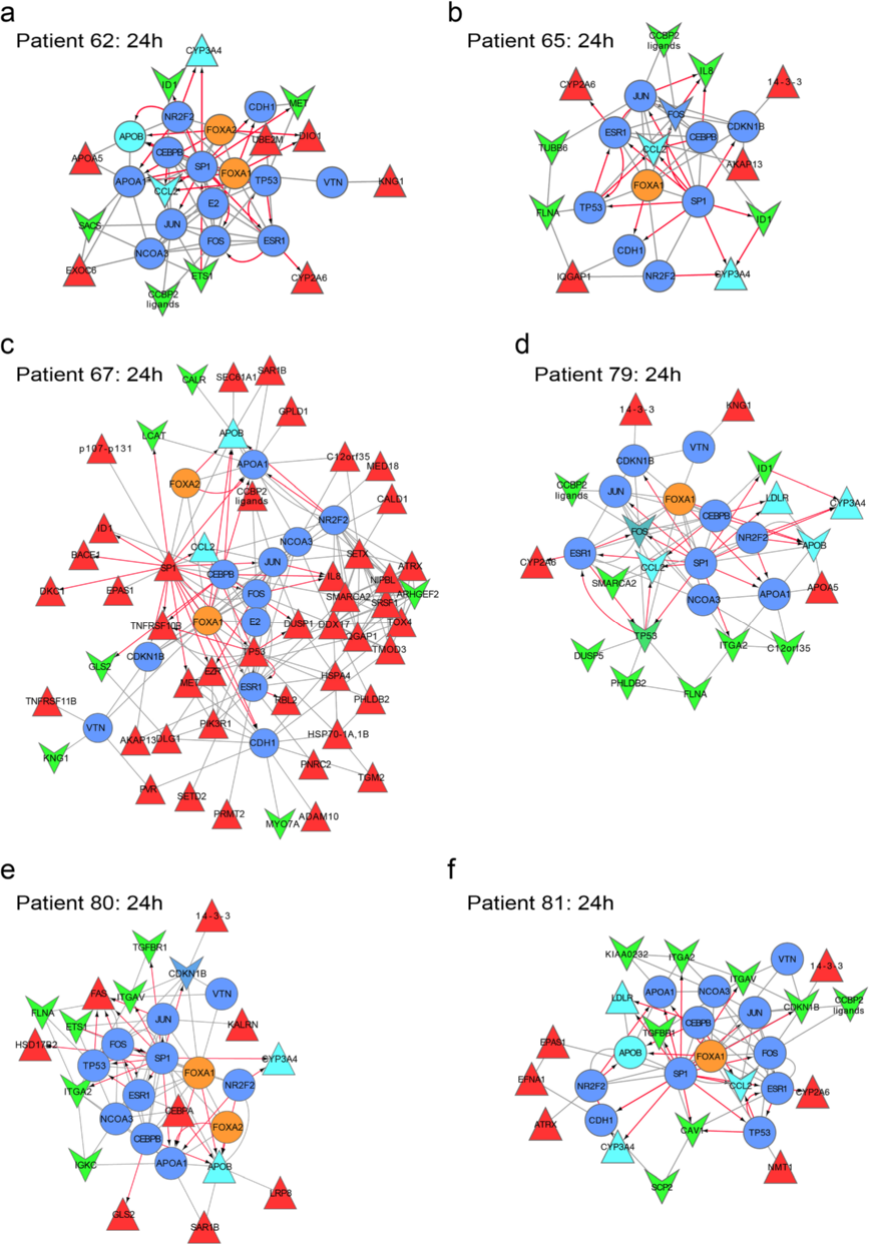
Neighbor focus networks of FoxA1 in all six patients at 24 h. We chose to explore the environment of FoxA1 with respect to differential expression. FoxA1 neighbor focus networks were created for patients 62 (**a**), 65 (**b**), 67 (**c**), 79 (**d**), 80 (**e**) and 81 (**f**). Up regulated nodes are shown as upward triangles, colored in red. Down regulated nodes are displayed as downward arrows, colored green. The Fox transcription factors are shown in orange, targets of atorvastatin are highlighted in cyan. Protein-protein interactions have grey edges; edges of transcription factor gene interactions are colored red

We chose one of the Fox transcription factors, FoxA1, as the start node and used a search depth of two. We assumed that an environment search with FoxA1 might shed light on the pathways and genes regulated by this transcription factor during atorvastatin treatment. We chose the 24 h time point for further analysis. Earlier time points showed only few differentially expressed genes for most of the patient samples. In later time points, a considerable amount of genes was changed, possibly due to secondary effects. Data from the six patients at time point 24 h are shown in Fig. 4.

The striking difference of patient 67 is again visible: the environment search of FoxA1 under the chosen conditions (2 steps, log2 fold change of at least 1.5) produced the largest network of all patients. Most proteins in patient 67’s network are up regulated. Notably, the central transcription factor Sp1 is up regulated, which is potentially the cause of the strong effect we observed in gene expression.

Many of the transcription factors that were present in the HMGCR-FoxA1/2/3 focus network are also present in the neighbor focus networks of the six patients. In all networks, Sp1 seems to be a central hub. The atorvastatin targets Ccl2 and Cyp3A4 are present in all but one network (Patient 80), while HMGCR is not found in any network. Note also that in Patient 79, most of the genes are down-regulated, including the transcriptional regulators Tp53 and Fos. Finally, we noticed that most nodes that are shared between all patients are linker nodes and thus not differentially expressed. Exceptions thereof are Sp1, Fos and Tp53, all of which show differential expression in at least one sample.

We decided to cluster the patient data based on the differential regulation and presence or absence of nodes in the FoxA1 neighbor focus network using Cluster3 [32]. The differential behavior of patient 67 is reflected in the resulting hierarchical tree (Additional file 4: Figure S3 a, data available in Additional file 1: Table S12). Furthermore, we observe a close clustering of patients 80 and 81, as well as a sub-group formed by patients 79, 65 and 62. We were interested, if the same groups would cluster at the 24 h time point in overall gene expression as well. We therefore used the expression values of the probe IDs at 24h (Additional file 1: Table S1) for cluster3 analysis (Additional file 4: Figure S3 b). Indeed, the clustering of all expression values is highly comparable; however, at the level of all analyzed genes, patient 62 displays more similarity to patient 79 than 65 in this small sub-group.

### Comparison to previous analysis

We started with the same primary data, as well as clustering technique as Schröder, et al. [20], resulting in identical clusters of co-regulated genes. Yet, our approach has channeled the analysis of the data in a different direction. In the previous study, enrichment analysis, followed by *Cis*-regulatory module detection in combination with network analysis was done. This yielded a set of transcription factors with a hypothetical function in atorvastatin-induced gene regulation. Factors such as Krüppel-like factors Klf4 and Klf11, hypoxia-inducible factor 1 (Hif1A), Hnf4, the nuclear receptor RXR in combination with other nuclear receptors, nuclear receptors PPARA, NR1H2 and NR2C2, Sp3 and Sp1, as well as Tgif1, Smad2 or Elf1 were found in [20]. In our primary atorvastatin focus network, we also found the transcription factors RXRB, Hnf4A, PPARA, NR2C2, Sp1, Sp3 and Klf4. The corresponding pathways of these transcription factors were likewise enriched (see Additional file 1: Tables S8 and S9). In the second step, however, we did not pursue any of the above factors or pathways. We rather focused on the FoxA transcription factors and their potential role in atorvastatin response. Interestingly, we also found Sp1 as a major transcriptional regulator in our focus networks. Sp1 is known to coordinate expression together with both, RXR/RAR (see for instance [50–53]), as well as the FoxA transcription factors [54–56]. Both, Sp1, as well as the FoxA transcription factors are also known to regulate some direct atorvastatin drug targets [36, 38, 39, 52, 53, 57].

viPEr was in this study used to point towards less well known players in atorvastatin response. In fact, we see the advantage of a plug-in such as viPEr in exploring paths that are difficult to find otherwise. If used with numerical values from experimental studies such as an expression screen, it will open up new avenues for experimental research.

## Conclusions

Here we present the Cytoscape plug-ins viPEr (virtual pathway explorer) and PEANuT (pathway analysis and enrichment tool). viPEr provides the possibility to navigate large interaction networks by linking two nodes or two groups of nodes with each other. It can be used to identify potential links between processes or pathways. viPEr furthermore enables users to explore the neighborhood of a single node with respect to the (numerical) quality of radiating paths. The Cytoscape plug-in PEANuT identifies enriched pathways in a focus network compared to a background network. We used viPEr to create focus networks, as well as neighbor focus networks using time-series data on atorvastatin-treated, primary hepatocytes from six donors. The focus network was analyzed using PEANuT. We identified the FoxA1/A2/A3 transcription factors to be involved in atorvastatin response. Furthermore, PEANuT revealed that the FoxA networks were enriched in cancer, as well as metabolic pathways. We found interesting differences in patient samples in the focus and neighbor focus networks, possibly explaining the often-observed, individual responses to drug treatment.

While we used viPEr with numerical values from a differential expression study, the plug-in can be used with numbers inferred from any experimental set-up and high-throughput assay. Functions ‘**A to B**’ and ‘**connecting in batch**’ work also without numerical values to create focus networks. In this case, no scoring of paths is done. The ‘**environment search**’, however, requires numerical values for the creation of a neighbor focus network.

## Availability and requirements

viPEr and PEANuT are all available via sourceforge.org. Both plug-ins have been tested for the versions 2.8.2 and 2.8.3 of Cytoscape. Versions of viPEr and PEANuT are also available for Cytoscape 3.2 and higher (viPEr for Cytoscape version 3 is available via http://sourceforge.net/projects/viperplugin/ and PEANuT for Cytoscape 3.2 and higher is available via http://sourceforge.net/projects/peanutv3/; for both apps, the source code is also available via sourceforge.net and both apps will be released via the Cytoscape 3 app manager). Please note that the current version of PEANuT v3 does not work with Cytoscape versions below 3.0 or 3.1. viPEr and PEANuT are fully integrated in both Cytoscape versions and compatible with other Cytoscape plug-ins. viPEr and PEANuT will be maintained to work with future releases of Cytoscape, as well as updates of public pathway database.

The required amount of memory depends mainly on the background network. In general, we advise to have at least 8 GB of RAM for the mouse or human interactome. We furthermore advise to work with turned-off view of large background networks (using the built-in Cytoscape function ‘destroy view’). Cytoscape itself is written in Java and runs on Linux, MacOS and Windows systems. We advise to use Oracle Java, as we have not extensively tested and validated our software for Open JDK.

## Additional files

Additional file 1: Table S1. log2 fold change of six selected patients at time points 6 h, 12 h, 24 h, 48 h and 72 h. Data were taken from [20], Affymetrix probe IDs are shown. **Table S2.** Time-course and patient- dependent clustering of probeIDs that showed differential expression in at least one time point or patient. 24 clusters were created using EDISA 3D clustering, containing 823 non-redundant, and differentially expressed genes. **Table S3.** Annotation of the EDISA 3D-selected, differentially expressed set of Affymetrix probe IDs. **Table S4.** GO enrichment of EDISA 3D-selected, differentially expressed set of Affymetrix probe IDs. **Table S5.** Pathway enrichment of EDISA 3D-selected, differentially expressed set of Affymetrix probe IDs. **Table S6.** Targets of atorvastatin as identified by STITCH. **Table S7.** GO enrichment of the atorvastatin focus network. **Table S8.** Pathway enrichment of atorvastatin focus network using PEANuT. **Table S9.** Pathway enrichment of the atorvastatin focus network using DAVID. **Table S10.** Pathway enrichment of the FoxA1/A2/A3 – HMGCR neighbor focus network using PEANuT. **Table S11.** Pathway enrichment of the FoxA1/A2/A3 – HMGCR neighbor focus network using DAVID. **Table S12.** Node scores for FoxA1 neighbor focus networks of the six patients used for clustering. (XLSX 5773 kb) Additional file 2: Figure S1. Focus network of all atorvastatin targets and differentially expressed genes after EDISA 3D clustering (see text). Up regulated nodes are shown as upward triangles, colored in red. Down regulated nodes are displayed as downward arrows, colored green. Protein-protein interactions have grey edges; edges of transcription factor gene interactions are colored red. (PDF 489 kb) Additional file 3: Figure S2. Focus networks of HMGCR and FoxA1/A2/A3 of the six patients. Differential expression of nodes at 12 h is highlighted. Data are shown for patients 62 (**a**), 65 (**b**), 67 (**c**), 79 (**d**), 80 (**e**) and 81 (**f**). Up regulated nodes are shown as upward triangles, colored in red. Down regulated nodes are displayed as downward arrows, colored green. The Fox transcription factors are shown in orange, as is the HMGCR. Protein-protein interactions have grey edges; edges of transcription factor gene interactions are colored red. (PDF 160 kb) Additional file 4: Figure 3. (**a**) Clustering of patients based on the FoxA1 neighbor focus network shown in Fig. 4, as well as the probe ID data from all patients at 24 h (**b**), data were taken from Additional file 1: Table S1. (PDF 75 kb)

## Abbreviations

PPI: Protein-Protein interaction
TFGI: Transcription Factor-Gene Interaction
HMGCR: HMG-CoA (3-hydroxy-3-methylglutaryl–coenzyme A) reductase
