# Supplementary figures and images for: Virtual pathway explorer (viPEr) and pathway enrichment analysis tool (PEANuT): creating and analyzing focus networks to identify cross-talk between molecules and pathways
Source: BMC Genomics. 2015 Oct 14;16:790. doi: 10.1186/s12864-015-2017-z (PMC4606501; doi:10.1186/s12864-015-2017-z)

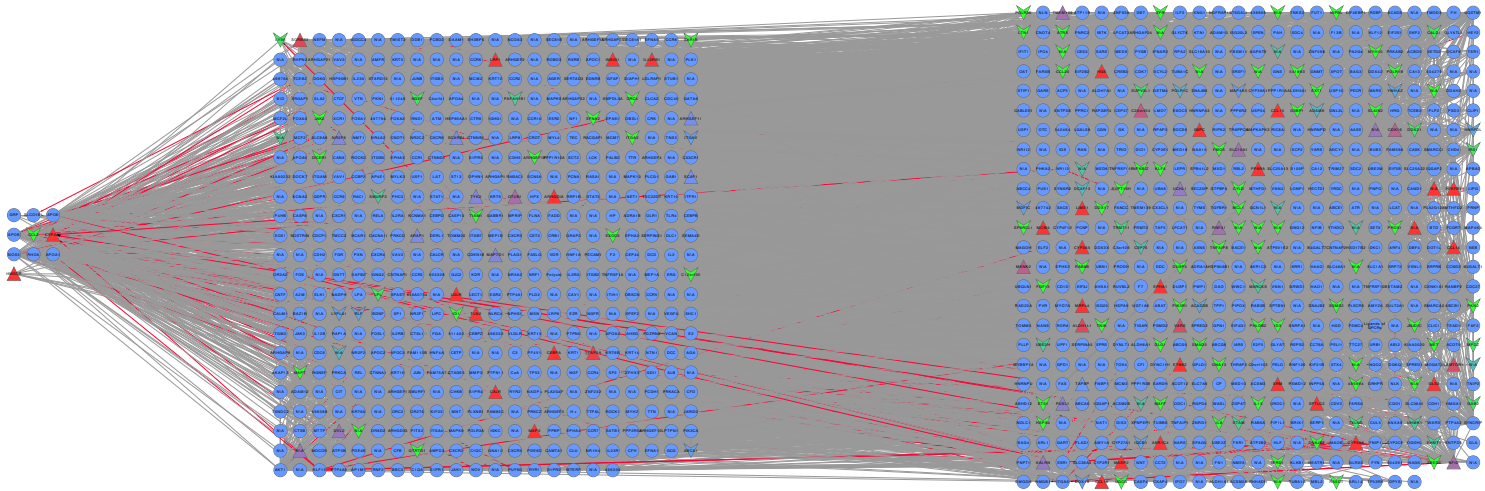

Supplement: Additional file 2: Figure S1. — Focus network of all atorvastatin targets and differentially expressed genes after EDISA 3D clustering (see text). Up regulated nodes are shown as upward triangles, colored in red. Down regulated nodes are displayed as downward arrows, colored green. Protein-protein interactions have grey edges; edges of transcription factor gene interactions are colored red. (PDF 489 kb) [file 12864_2015_2017_MOESM2_ESM.pdf]

a

Patient 62

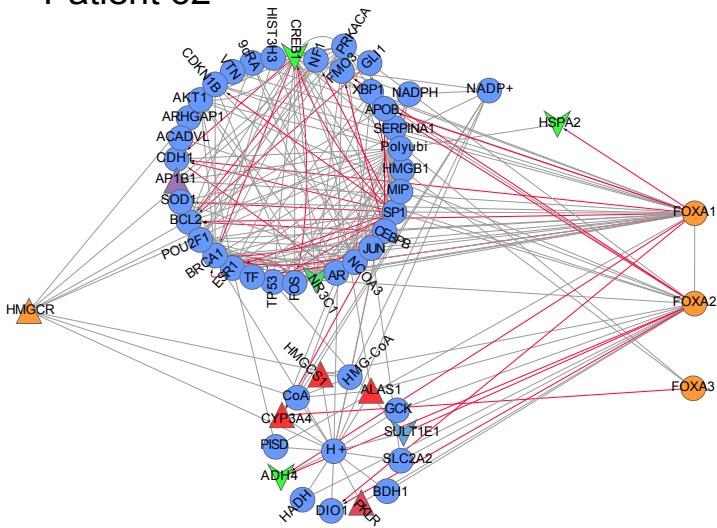

b

Patient 65

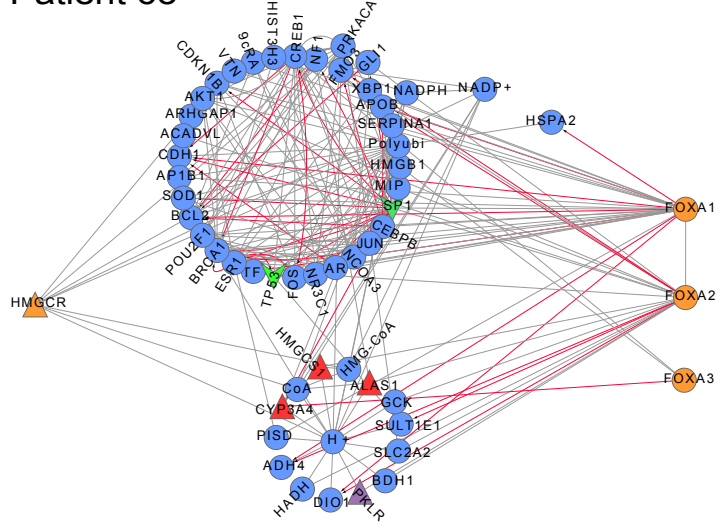

c

Patient 67

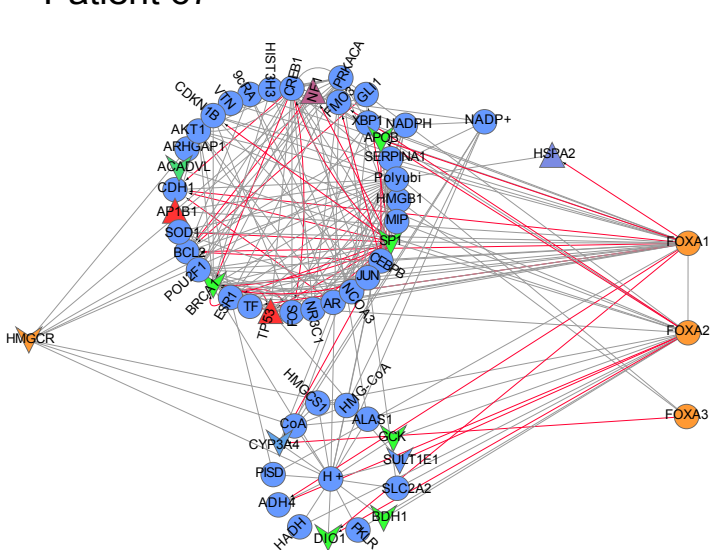

d

Patient 79

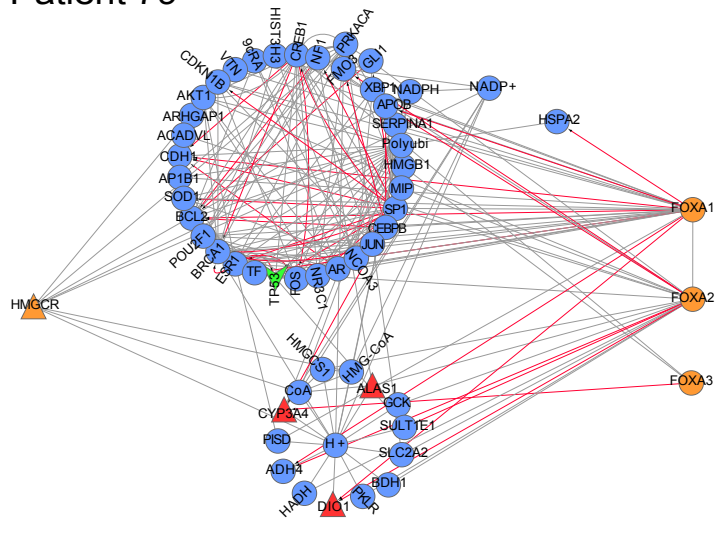

e

Patient 80

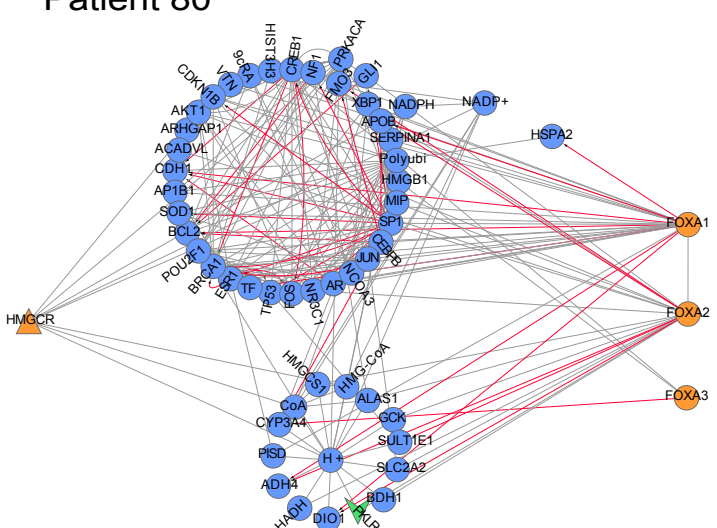

f

Patient 81

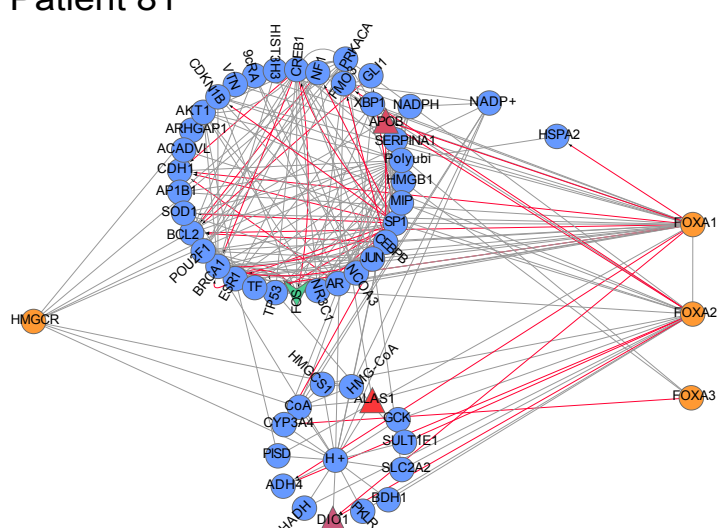

Supplement: Additional file 3: Figure S2. — Focus networks of HMGCR and FoxA1/A2/A3 of the six patients. Differential expression of nodes at 12 h is highlighted. Data are shown for patients 62 (a), 65 (b), 67 (c), 79 (d), 80 (e) and 81 (f). Up regulated nodes are shown as upward triangles, colored in red. Down regulated nodes are displayed as downward arrows, colored green. The Fox transcription factors are shown in orange, as is the HMGCR. Protein-protein interactions have grey edges; edges of transcription factor gene interactions are colored red. (PDF 160 kb) [file 12864_2015_2017_MOESM3_ESM.pdf]

a

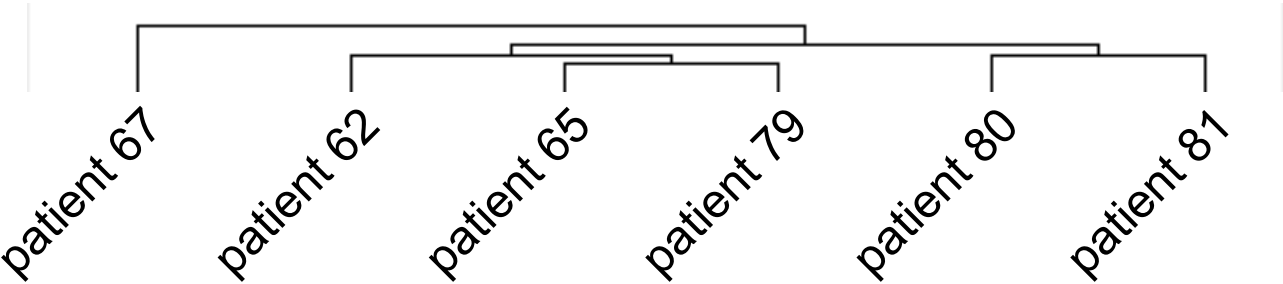

b

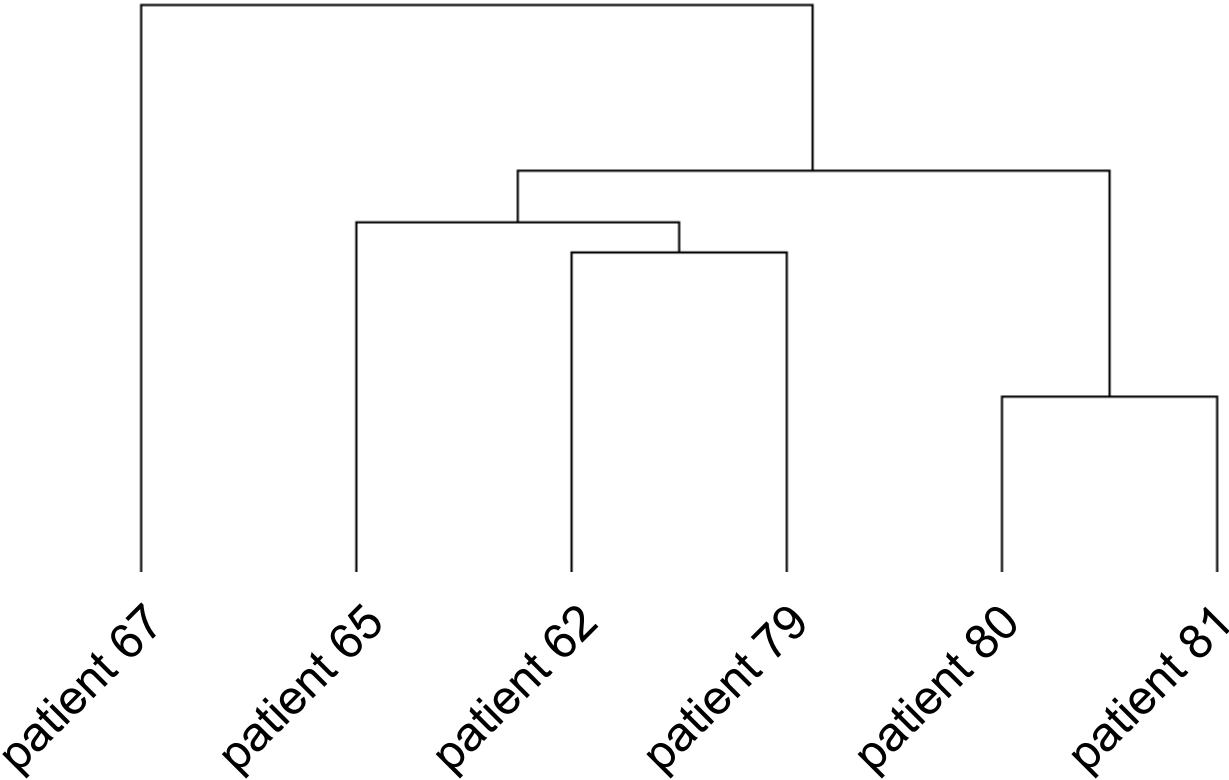

Supplement: Additional file 4: Figure 3. — (a) Clustering of patients based on the FoxA1 neighbor focus network shown in Fig. 4, as well as the probe ID data from all patients at 24 h (b), data were taken from Additional file 1: Table S1. (PDF 75 kb) [file 12864_2015_2017_MOESM4_ESM.pdf]
